# The motor repertoire in 3- to 5-month old infants with Down syndrome

**DOI:** 10.1016/j.ridd.2017.05.006

**Published:** 2017-08

**Authors:** Dafne Herrero, Christa Einspieler, Carolina Y. Panvequio Aizawa, Akmer Mutlu, Hong Yang, Alice Nogolová, Jasmin Pansy, Karin Nielsen-Saines, Peter B. Marschik, GenGM Study Group

**Affiliations:** a School of Public Health, University of São Paulo, São Paulo, Brazil; b Research Unit iDN, Interdisciplinary Developmental Neuroscience, Department of Phoniatrics, Medical University of Graz, Graz, Austria; c Department of Physical Therapy, Communication Science & Disorders and Occupational Therapy, School of Medicine, University of São Paulo, São Paulo, Brazil; d Developmental and Early Physiotherapy Unit, Department of Physiotherapy and Rehabilitation, Faculty of Health Sciences, Hacettepe University, Ankara, Turkey; e Rehabilitation Department, Children’s Hospital of Fudan University, Shanghai, China; f Children’s Department, City Hospital of Ostrava, Ostrava, Czech Republic; g Division of Neonatology, Department of Pediatrics and Adolescent Medicine, Medical University of Graz, Graz, Austria; h Division of Infectious Diseases, David Geffen School of Medicine, University of California Los Angeles, Los Angeles, CA, USA; i Center of Neurodevelopmental Disorders (KIND), Department of Women’s and Children’s Health, Karolinska Institutet, Stockholm, Sweden

**Keywords:** Down syndrome, Fidgety movements, General movements, Infant, Motor optimality score

## Abstract

**Background:**

Even though Down syndrome is the most common chromosomal cause of intellectual disability, studies on early development are scarce.

**Aim:**

To describe movements and postures in 3- to 5-month-old infants with Down syndrome and assess the relation between pre- and perinatal risk factors and the eventual motor performance.

**Methods and procedures:**

Exploratory study; 47 infants with Down syndrome (26 males, 27 infants born preterm, 22 infants with congenital heart disease) were videoed at 10–19 weeks post-term (median = 14 weeks). We assessed their Motor Optimality Score (MOS) based on postures and movements (including fidgety movements) and compared it to that of 47 infants later diagnosed with cerebral palsy and 47 infants with a normal neurological outcome, matched for gestational and recording ages.

**Outcomes and results:**

The MOS (median = 13, range 10–28) was significantly lower than in infants with a normal neurological outcome (median = 26), but higher than in infants later diagnosed with cerebral palsy (median = 6). Fourteen infants with Down syndrome showed normal fidgety movements, 13 no fidgety movements, and 20 exaggerated, too fast or too slow fidgety movements. A lack of movements to the midline and several atypical postures were observed. Neither preterm birth nor congenital heart disease was related to aberrant fidgety movements or reduced MOS.

**Conclusions and implications:**

The heterogeneity in fidgety movements and MOS add to an understanding of the large variability of the early phenotype of Down syndrome. Studies on the predictive values of the early spontaneous motor repertoire, especially for the cognitive outcome, are warranted.

**What this paper adds:**

The significance of this exploratory study lies in its minute description of the motor repertoire of infants with Down syndrome aged 3–5 months. Thirty percent of infants with Down syndrome showed age-specific normal fidgety movements. The rate of abnormal fidgety movements (large amplitude, high/slow speed) or a lack of fidgety movements was exceedingly high. The motor optimality score of infants with Down syndrome was lower than in infants with normal neurological outcome but higher than in infants who were later diagnosed with cerebral palsy. Neither preterm birth nor congenital heart disease were related to the motor performance at 3–5 months.

## 1 Introduction

Even though Down syndrome is the most common chromosomal cause of intellectual disability, with 20–22 individuals per 10,000 births affected (e.g., Kurtovic-Kozaric et al., 2016, Loane et al., 2013), studies on early development are scarce. Infants with Down syndrome are known to be socially competent but show a delay in the acquisition of motor milestones and deficits in early gesture production (Grieco, Pulsifer, Seligsohn, Skotko, & Schwartz, 2015; Özcaliskan, Adamson, Dimitrova, Bailey, & Schmuck, 2016; Saito & Watanabe, 2016). As early as the first months of life they scored lower than typically developing infants on both the Test of Infant Motor Performance (Cardoso, Campos, Santos, Santos, & Rocha, 2015) and the Alberta Infant Motor Scale (Tudella, Pereira, Pedrolongo Basso, & Savelsbergh, 2011). They kicked less often (Ulrich & Ulrich, 1995) and their arm movements were less accurate when reaching for objects of different sizes (de Campos, Cerra, Silva, & Rocha, 2014). Repeated assessments of their spontaneous general movements revealed a heterogeneous movement quality, although the fluency and complexity tended to improve between 1 and 6 months of age (Mazzone, Mugno, & Mazzone, 2004).

Initially designed for infants with acquired brain injuries, the Prechtl assessment of general movements (Einspieler & Prechtl, 2005; Prechtl et al., 1997) has recently also been applied to infants with genetic syndromes (Einspieler, Hirota, Yuge, Deijima, & Marschik, 2012; Einspieler, Kerr, & Prechtl, 2005; Einspieler et al., 2014; Marschik, Soloveichick, Windpassinger, & Einspieler, 2015; Mazzone et al., 2004) and infants later diagnosed with autism spectrum disorders (Einspieler et al., 2014, Zappella et al., 2015). The assessment is based on visual Gestalt perception of normal vs. abnormal movements in the entire body (i.e. general movements). It is applied in foetuses, preterm infants, and newborn infants from term to 5 months post-term (Einspieler, Prechtl, Bos, Ferrari, & Cioni, 2004; Prechtl & Einspieler, 1997). The excellent predictive power of general movement assessments (Bosanquet, Copeland, Ware, & Boyd, 2013; Einspieler et al., 2004) is mainly attributable to fidgety general movements, which occur from 3 to 5 months post-term age (Einspieler & Prechtl, 2005; Prechtl et al., 1997). Infants with normal fidgety movements are very likely to develop normally in neurological terms, whereas infants who never develop fidgety movements have a high risk for neurological impairment (Einspieler & Prechtl, 2005; Prechtl et al., 1997). Adding a detailed assessment of concurrent movements and postures to the assessment of fidgety movements, for example, showed a reduced motor optimality score (MOS) to be associated with a limited activity in children who were later diagnosed with cerebral palsy (Yang et al., 2012), or with lower intelligent quotients during school age (Butcher et al., 2009). We therefore assumed that determining the MOS (Einspieler et al., 2004, p. 26) by assessing fidgety movements as well as concurrent movement and postural patterns would enable us to systematically document the motor repertoire of infants with Down syndrome. The MOS makes it possible to quantitatively relate pre- and perinatal data to the motor repertoire of an infant and to data obtained from follow-up studies.

The aims of our study were (1) to describe movements and postures in 3- to 5-month-old infants with Down syndrome; (2) to compare their MOS with the MOS of two matched samples, one of which was later diagnosed with cerebral palsy, while the other had a normal neurological outcome; and (3) to analyse to what extent clinical risk factors during pregnancy, at delivery, and during the neonatal period were related to the motor performance in 3- to 5-month-old infants with Down syndrome.

## 2 Methods

### 2.1 Participants

This exploratory study comprised a convenience sample of 47 infants with Down syndrome − 21 females (45%) and 26 males (55%) − who had been admitted to (a) the Darcy Vargas Public Hospital, São Paulo (17 individuals); (b) the Department of Physiotherapy and Rehabilitation at the Hacettepe University in Ankara (ten individuals); (c) the Associação de Pais e Amigos dos Excepcionais at São Paulo University (seven individuals); (d) the Rehabilitation Department of the Children’s Hospital of Fudan University in Shanghai (four individuals); (e) the Children’s Department at the City Hospital of Ostrava (three individuals); (f) the Clinic of Early Intervention at the University Hospital São Paolo (three individuals); and (g) the Medical University of Graz (three individuals) between June 2015 and May 2016. In order for the infants to be included in the study, the infants’ motor performance had to be recorded between 9 and 20 weeks post-term. The infants’ gestational ages at birth ranged from 29 to 41 weeks (median = 37 weeks), with a birth weight range of 1440 g to 3680 g (median = 2585 g). Twenty-seven infants were born preterm (57%), including two monozygotic twin pairs. Other clinical characteristics obtained from the medical histories are presented in Table 1. Three infants were diagnosed with mosaic Down syndrome, one with Robertsonian translocation (14;21).

**Table 1:** Clinical characteristics of 47 infants with Down syndrome.

	Median, Interquartile (P_25_, P_75_) and Range; or N (%)
Maternal age	Median = 35 years P_25_ = 31 years; P_75_ = 39 years; range: 17–45 years
Paternal age	Median = 37 years P_25_ = 32 years; P_75_ = 42 years; range: 23–50 years
Parity	Median = 2 P_25_ = 1; P_75_ = 3; range: 1–5
Pregnancy complications	22 (47%)
Preterm birth	27 (57%)
Caesarean section	31 (66%)
Birth weight	Median = 2585 g P_25_ = 2000 g; P_75_ = 2980 g; range: 1400–3680 g
Apgar Score at 1 min	Median = 8 P_25_ = 7; P_75_ = 8; range: 6–10
Apgar Score at 5 min	Median = 9 P_25_ = 8; P_75_ = 9; range: 8–10
Hyperbilirubinaemia	26 (55%)
CHD: (Atrio)ventricular septal defects and/or patent ductus arteriosus	22 (47%)
Duration of mechanical ventilation	Median = 0 days P_25_ = 0 days; P_75_ = 3 days; range: 0–19 days
Age at discharge from the hospital	Median = 12 days P_25_ = 4 days; P_75_ = 30 days; range: 3–90 days

Key: CHD, congenital heart disease.

For comparison we used data of our international, MOS-based data bank (N = 365 as of January 15, 2017), picking (i) 47 individuals with a normal neurological outcome at 3–5 years of age, whose gestational age and age of video recording matched our study cases; and (ii) 47 individuals with a comparable gestational age at birth and post-term age at the time of the video recording who were diagnosed with cerebral palsy at 3–5 years of age (Table 2). Since spontaneous (i.e. endogenously generated) movements are not related to ethnicity (Luxwolda et al., 2014), we did not match the ethnic background.

**Table 2:** The motor optimality score (MOS) and its subcategories in 47 infants diagnosed with Down syndrome, 47 infants diagnosed with cerebral palsy, and 47 infants with a normal neurological outcome at 3 to 5 years of age.

	Down Syndrome (DS)	Cerebral Palsy (CP)	Normal Neurological Outcome (N)	p-values
Preterm birth	27 (57.4%)	27 (57.4%)	27 (57.4%)	
Recording age				
9–12 weeks	12 (25.5%)	16 (34%)	12 (25.5%)	
13–16 weeks	30 (64%)	24 (51%)	30 (64%)	
17–20 weeks	5 (10.5%)	7 (15%)	5 (10.5%)	
MOS	Median = 13	Median = 6	Median = 26	DS vs. CP
	P_25_ = 12	P_25_ = 6	P_25_ = 21	p < 0.01^a^
	P_75_ = 23	P_75_ = 8	P_75_ = 28	DS vs. N
	Range: 10–28	Range: 5–20	Range: 10–28	p < 0.01^a^
Fidgety movements				DS vs. CP
Normal	14 (30%)	1 (2%)	44 (93.5%)	p < 0.01^b^
Abnormal^x^	20 (42.5%)	0 (0%)	0 (0%)	DS vs. N
Absent	13 (27.5%)	46 (98%)	3 (6.5%)	p < 0.01^b^
Repertoire				DS vs. CP
Age-adequate	12 (25.5%)	0 (0%)	27 (57.5%)	p < 0.01^b^
Reduced	20 (42.5%)	4 (8.5%)	15 (32%)	DS vs. N
Not age-adequate	15 (32%)	43 (91.5%)	5 (10.5%)	p < 0.01^b^
Movements				DS vs. CP
N > A	39 (83%)	6 (12.5%)	36 (76.5%)	p < 0.01^b^
N = A	5 (10.5%)	11 (23.5%)	8 (17%)	DS vs. N
N < A	3 (6.5%)	30 (64%)	3 (6.5%)	p = 0.67^b^
Posture				DS vs. CP
N > A	22 (47%)	8 (17%)	31 (66%)	p < 0.01^b^
N = A	10 (21%)	5 (10.5%)	6 (13%)	DS vs. N
N < A	15 (32%)	34 (72.5%)	10 (21%)	p = 0.17^b^
Movement character				DS vs. N
Smooth, fluent	3 (6.5%)	0 (0%)	19 (40.5%)	p < 0.01^b^
Abnormal, not CS	44 (93.5%)	34 (72%)	28 (59.5%)	DS vs. CP
CS	0 (0%)	13 (28%)	0 (0%)	p < 0.01^b^

Key: N > A, more normal than abnormal patterns; N = A, an equal number of normal and abnormal patterns; N < A, fewer normal than abnormal patterns; CS, cramped-synchronised movement character, i.e. spontaneous general movements appear stiff; limb and trunk muscles contract almost simultaneously and then relax almost simultaneously (Einspieler et al., 2004).

a Mann-Whitney-*U* test.

b Pearson Chi-square test.

x this category also includes infants with fidgety-like movements (large amplitude, slow).

All parents gave their written informed consent. The ethical review boards of the various centres approved the study.

### 2.2 Recording and evaluation of movements and postures at 3–5 months post-term age

Within the GenGM network we recorded 5-min videos of the spontaneous motility of each infant at a median post-term age of 14 weeks (P_25_ = 12 weeks; P_75_ = 15 weeks; range: 10–19 weeks). The recordings were performed during periods of active wakefulness between feedings, with the infant partly dressed, lying in supine position (Einspieler et al., 2004). The videos were evaluated by at least two certified raters (D.H., C.E., A.M., A.N., J.P., P.B.M.) according to the Prechtl method of global and detailed general movement assessment (Einspieler et al., 2004 Einspieler & Prechtl, 2005). Scorers C.E. and P.B.M. were not familiar with the details of the participants’ clinical histories apart from the fact that they had Down syndrome. In case of disagreement (four recordings; 8.5%), the raters re-evaluated the recordings until consensus was reached on a final score.

Fidgety movements and the concurrent repertoire of movements and postures were assessed independently in separate runs of the video recordings. Using the score sheet for the assessment of motor repertoire at 3–5 months (Einspieler et al., 2004, p. 26), we calculated the MOS, with a maximum value of 28 (for the best possible performance) and a minimum value of 5. The score sheet comprises the following five sub-categories: (i) fidgety movements, (ii) age-adequacy of motor repertoire, (iii) quality of movement patterns other than fidgety movements, (iv) posture, and (v) overall quality of the motor repertoire (Einspieler et al., 2004, Yuge et al., 2011). Fjørtoft and colleagues found a high inter-observer reliability for the MOS with intra-class correlation coefficients ranging from 0.80 to 0.94 (Fjørtoft, Einspieler, Adde, & Strand, 2009).

### 2.3 Statistical analysis

Statistical analysis was performed using the SPSS package for Windows, version 23.0 (SPSS Inc., Chicago, IL). The Pearson Chi-square test was used to evaluate associations between nominal data. To put the medians of non-normally distributed continuous data (e.g. motor optimality score) in relation to nominal data (e.g. preterm birth), we applied the Mann-Whitney-U test or, if there were more than two categories, the Kruskal-Wallis test (e.g. repertoire). To assess the relative strength of the association between variables, we computed the following correlation coefficients: Cramer’s V coefficient was applied when at least one of the two variables was nominal (e.g. preterm birth and age-adequacy of the repertoire). To assess the relation between two continuous variables (e.g. gestational age and motor optimality score), we applied the Pearson product-moment correlation coefficient. Throughout the analyses, p < 0.05 (two-tailed) was considered to be statistically significant.

## 3 Results

### 3.1 The motor performance of infants with Down syndrome at 3–5 months postterm age (Table 2, first column)

Fourteen infants had normal fidgety movements (30%); six infants (12.5%) displayed abnormal fidgety movements (i.e. they look like normal ones though with a greater amplitude, speed and jerkiness); 13 infants (27.5%) displayed no fidgety movements, which were therefore classified as absent; 14 infants (30%) showed fidgety-like movements whose amplitude was too great and whose pace was too slow. Abnormal and absent fidgety movements as well as fidgety-like movements were grouped as aberrant fidgety movements (33 infants; 70%).

Twelve infants (25.5%) displayed an age-adequate movement repertoire. The repertoire was found to be reduced in 20 infants (42.5%; mainly due to a lack of movements to the midline), and age-inadequate in 15 infants (32%).

The quality of the various movement patterns (other than fidgety movements) was scored as predominantly normal in 39 infants (83%) and predominantly abnormal in three infants (6.5%); five infants (10.5%) showed an equal number of normal and abnormal movements. On average the infants demonstrated three normal movement patterns (range: 0–8) and one abnormal movement pattern (range: 0–3). The most frequent normal movement patterns included visual scanning (32/47; 68%), side-to-side movements of the head (22/47; 47%), foot-to-foot contact (14/47; 30%), hand-to-mouth contact (12/47; 25.5%), and kicking (10/47; 21%). Smiling (9/47; 19%), fiddling (9/47; 19%), hand regards (8/47; 17%), swipes (7/47; 15%), hand-to-hand contact (6/47; 13%), arching (6/47; 13%), and leg lifting (5/47; 11%) were observed in fewer than ten individuals. Other movement patterns such as wiggling-oscillating arm movements, hand-to-knee contact, or rolling to the side were observed in fewer than five individuals (<10%). The most frequent abnormal movement pattern was long-lasting and/or repetitive tongue protrusion (26/47; 55%). In a few infants we observed hand-to-hand contact with no mutual manipulation (4/47; 8.5%), long lasting wiggling-oscillating arm movements (3/47; 6%), repetitive kicking (3/47; 6%), and monotonous side-to-side movements of the head (1/47; 2%).

Posture was rated as predominantly normal in 22 infants (47%) and predominantly abnormal in 15 infants (32%); ten infants (21%) showed an equal number of normal and abnormal postures (Table 2). Infants with predominantly normal postural patterns were able to hold their head in midline (31/47; 66%), showed a symmetrical body posture (25/47; 53%) and variable finger postures (23/47; 49%); a persistent asymmetric tonic neck response was absent in all individuals. On average the infants demonstrated three normal postural patterns (range: 1–4) and two abnormal postural patterns (range: 0–7). The most common abnormal pattern was a lack of variable finger postures (24/47; 51%) with just a few monotonous finger postures, finger spreading and/or predominant fisting. Twelve infants (25.5%) kept both arms predominantly extended, while seven infants (15%) kept their legs extended most of the time. Hyperextension of the neck and trunk was seen in three individuals (6.5%). The following two postural atypicalities were observed, but are not captured by the MOS sheet: nine individuals (19%) showed an internal rotation and pronation of one or both wrists, and 22 infants (47%) showed an external rotation and abduction of the hips (which was also the reason why most of them were unable to show foot-to-foot contact).

Only three infants (6.5%) exhibited a normal, smooth and fluent overall movement character, while 44 infants (93.5%) displayed a monotonous, stiff, jerky and/or tremulous movement character.

The median MOS was 13 (P_25_ = 12; P_75_ = 23; range: 10–28).

### 3.2 The MOS of infants with Down syndrome compared to infants later diagnosed with cerebral palsy and infants with a normal neurological outcome (Table 2)

The MOS of infants with Down syndrome was significantly lower than that of infants with a normal neurological outcome (p < 0.01) but significantly higher than that of infants later diagnosed with cerebral palsy (p < 0.01). Similar results were obtained for fidgety movements, the age-adequacy of the motor repertoire, and the overall movement character (*p*-values < 0.01). The quality of movement and postural patterns of infants with Down syndrome were similar to those of infants with a normal neurological outcome (*p*-values > 0.10), while infants later diagnosed with cerebral palsy scored lower (*p*-values < 0.01; Table 2).

### 3.3 The neonatal period and its relation to the motor performance at 3–5 months post-term age (Table 3, Table 4)

None of the clinical characteristics was related to the MOS or fidgety movements. Preterm birth was not associated with the MOS or its subcategories (Table 3), nor was any other clinical variable. We would particularly like to mention that congenital heart disease (CHD) was not related to the motor performance at 3–5 months. Cranial ultrasound data were only available for a small proportion of the sample (eight infants, 17%). Two infants with abnormal cranial ultrasound findings had abnormal fidgety-like movements. Among the six infants with normal cranial ultrasound findings three showed normal fidgety movements, one infant did not develop fidgety movements, and two infants had slow abnormal fidgety-like movements ().

**Table 3:** The motor optimality score (MOS) and its subcategories in 47 infants diagnosed with Down syndrome according to preterm or term birth.

	Preterm Born n = 27	Term Born n = 20	p-values
MOS	Median = 13 P_25_ = 12 P_75_ = 24 Range: 10–26	Median = 13 P_25_ = 12 P_75_ = 16 Range: 10–28	p = 0.71^a^
Fidgety movements Normal Abnormal^x^ Absent	10 (37%) 10 (37%) 7 (26%)	4 (20%) 10 (50%) 6 (30%)	p = 0.65^b^
Repertoire Age-adequate Reduced Not age-adequate	4 (15%) 12 (44%) 11 (41%)	8 (40%) 8 (40%) 4 (20%)	p = 0.11^b^
Movements N > A N = A N < A	22 (81.5%) 4 (15%) 1 (3.5%)	17 (85%) 1 (5%) 2 (10%)	p = 0.41^b^
Posture N > A N = A N < A	14 (52%) 7 (26%) 6 (22%)	8 (40%) 3 (15%) 9 (45%)	p = 0.24^b^
Movement character Smooth, fluent Abnormal, not CS	2 (7.5%) 25 (92.5%)	1 (5%) 19 (95%)	p = 0.56^b^

Key: as in Table 2.

**Table 4:** Significant associations between clinical characteristics and specific motor patterns in 47 3- to 5-month-old infants with Down syndrome.

Caesarean section	Jerky movement character Cramer’s V = 0.32; p < 0.05 External rotation and abduction of hips Cramer’s V = 0.34; p < 0.05
Hyperbilirubinaemia	Jerky movement character Cramer’s V = 0.30; p < 0.05

Key: ^a^ Mann-Whitney-*U* test.

Table 4 only lists significant associations between clinical variables and items of the MOS. A jerky movement character (observed in 26/47 infants; 55%) was associated with caesarean section and hyperbilirubinaemia, although the two clinical variables were not related to each other (Pearson Chi-square test, p = 0.58). Delivery by caesarean section was also related to a higher occurrence of a particular atypical posture at 3–5 months: in many cases external rotation and abduction of the hips led to a lack of (especially lower limb) movements to the midline.

No difference was observed between twins and singletons (Fisher sign test, p > 0.05). Neither the three infants with mosaic Down syndrome (two with absent fidgety movements, one with normal fidgety movements; MOS = 10, 12 and 24, respectively) nor the one with Robertsonian translocation (normal fidgety movements, MOS = 23) showed any sort of specific features in their motor performance at 3–5 months post term.

## 4 Discussion

Apart from one individual reported in the context of a larger sample of high-risk infants in Japan (Yuge et al., 2011), this is the first study to use the MOS to describe fidgety movements and the concurrent motor repertoire in infants with Down syndrome. Mazzone et al. (2004) applied the detailed scoring for writhing general movements (observable from birth to 1–2 months post-term) up to the age of 6 months rather than for fidgety and concurrent motor patterns, which occur from 3 months onwards.

The motor coordination and performance of children, adolescents and adults with Down syndrome has been found to be highly heterogeneous (Latash, 2007). Interestingly, we already observed this heterogeneity at a much younger age: 30% of our 3- to 5-month-old infants with Down syndrome showed normal fidgety movements, 27.5% had no fidgety movements, and 42.5% showed abnormal fidgety movements. These highly diverse findings are reflected in the MOS, which range from 10 to the highest possible score of 28. Fifty percent of the infants scored between 10 and 13, which is significantly lower than infants with a normal neurological outcome and higher than infants who were later diagnosed with cerebral palsy. So far, two studies carried out in children with cerebral palsy in China, Italy, and the Netherlands demonstrated the lower the MOS at 3–5 months, the more severely limited their gross motor function (Bruggink et al., 2009, Yang et al., 2012). As we intend to monitor our sample of children with Down syndrome for at least 2 more years, we shall also assess the relation between the MOS and the motor, cognitive and language outcomes.

The one infant with Down syndrome described by Yuge et al. (2011) displayed abnormal fidgety movements and an MOS of 13. Abnormal fidgety movements, exaggerated in amplitude, speed and jerkiness, were also observed in six infants in the present study. This is an exceptionally high rate of occurrence, as abnormal fidgety movements are usually rare (see also Table 2). It has been a matter of debate whether or not infants with a low muscle tone are more likely to show abnormal fidgety movements (Einspieler, Peharz, & Marschik, 2016; Yuge et al., 2011). Although this may not be consentaneously defined, a low muscle tone is a general feature in infants with Down syndrome (Latash, 2007; Morris, Vaughan, & Vaccaro, 1982). A number of studies have documented an association between abnormal fidgety movements and coordination difficulties and/or disabilities in fine manipulative skills at school age (Einspieler et al., 2007, Einspieler et al., 2016); others describe an exceedingly high rate of abnormal fidgety movements in infants who were later diagnosed with autism spectrum disorders or Rett syndrome (Einspieler et al., 2005, Einspieler et al., 2014). However, most so-called abnormal fidgety movements in infants later diagnosed with autism spectrum disorders or Rett syndrome did not correspond with the category of abnormal fidgety movements described in infants with brain injuries, which were exaggerated in amplitude and speed (Einspieler & Prechtl, 2005; Einspieler et al., 2016, Prechtl et al., 1997). Several infants with a later diagnosis of autism spectrum disorders or Rett syndrome showed continual fidgety activity which was exaggerated in amplitude but too slow (Einspieler et al., 2005, Einspieler et al., 2014, Zappella et al., 2015). We also observed this pattern in 14/47 (30%) infants with Down syndrome (see the abnormal fidgety movements’ category in Table 2). It remains unclear whether this pattern is related to low muscle tone, another early atypicality in autism spectrum disorders (Flanagan, Landa, Bhat, & Bauman, 2012) and Rett syndrome (Nomura & Segawa, 1990), as video analysis does not allow for an assessment of active muscle strength or resistance to passive movements. Data on neurological examinations were not available in 44 of 47 individuals. In any case, the low rate of movements to the midline (i.e., foot-to-foot contact in 30% and hand-to-hand contact in only 13% of our sample) and the lack of kicking (21%) are in line with previous studies, where they have also been discussed as possible consequences of low muscle tone (Tudella et al., 2011). The same is true of the frequent external rotation and abduction of the hip (observed in 47% of infants with Down syndrome) as well as the uni- or bilateral internal rotation and pronation of the wrist(s) (19%). Unfortunately we are unable to confirm this association for lack of information about our infants’ muscle tone (see section 4.1.).

Several research groups have reported a significant impact of pre- and perinatal variables on the MOS at 3–5 months. For example, prenatal exposure to environmental pollutants or selective serotonin reuptake inhibitors, and perinatal hypoxic events resulted in a reduced MOS (Berghuis, Soechitram, Hitzert, Sauer, & Bos, 2013; de Vries, van der Veere, Reijneveld, & Bos, 2013; Yuge et al., 2011). No such association was established in our study. Neither gestational age nor birth weight or any other perinatal risk factor was found to be significantly related to the MOS or fidgety movements, notwithstanding the fact that 27 infants (57%) were born preterm. Nor was the percentage of aberrant fidgety movements and/or a lower MOS increased in the 22 infants with CHD, although toddlers with Down syndrome and CHD were reported to have a higher percentage of motor, language and cognitive deficits at 12–14 months than toddlers with Down syndrome and a normal heart structure (Alsaied et al., 2016, Visootsak et al., 2016). In our study, neither aberrant fidgety movements nor a reduced MOS were attributable to preterm birth or CHD.

Only two variables were shown to affect movements and postures at 3–5 months: caesarean section and hyperbilirubinaemia were related to a jerky movement character, and external rotation and abduction of hips was more common among infants born by caesarean section. In their study on healthy full-term infants during the first week after birth, Ploegstra, Bos, and de Vries (2014) compared the general movements of low-risk neonates born by vaginal delivery with those of neonates after caesarean section and found no difference. So far, we cannot explain how external rotation and abduction of the hips is related to caesarean section. As for the jerky movement character, there have no doubt been more revealing findings: Groen, de Blécourt, Postema, and Hadders-Algra (2005) reported 11 out of 15 children to have shown a normal neurological outcome in spite of jerky movements at the age of 2–4 months.

### 4.1 Limitations to our study

As various motor patterns such as abnormal fidgety or fidgety-like movements, lack of movements to the midline, and external rotation and abduction of the hips could be attributed to low muscle tone, we would also have needed to assess the infants’ muscle tone. Yet, the assessment of muscle tone is anything but unequivocal. Definitions are not standardised and inter-scorer agreement is prone to be low (Latash, 2007, Prechtl, 2001, Touwen, 1976) except for extremes. Diverging experience and procedures in the tonus assessment would no doubt have been additional challenges in a multicentre approach. Training and application of the Hammersmith Infant Neurological Examination (Haataja et al., 1999) was not available in most of the centres contributing to the study.

This being a comparative study, one might consider our participants’ varied ethnic backgrounds as problematic. However, as we are dealing with spontaneous, i.e. endogenously generated movements, the respective care-giving practices are very unlikely to have had an impact on the early motor patterns in question. In fact, general movements have been assessed worldwide for more than 20 years with similar cross-cultural results (e.g., Bruggink et al., 2009, Luxwolda et al., 2014, Yang et al., 2012, Yuge et al., 2011). Nor did the various sensory stimulations affect the infants’ fidgety movements, which demonstrates their robust, environment-independent character (Dibiasi & Einspieler, 2002; Dibiasi & Einspieler, 2004).

Of course, the infants’ different cultural backgrounds could be an issue with regard to the subcategory “posture”. Infants raised in a hammock, for example, seem to be faster in acquiring the “head in midline” position and/or a “symmetric body posture” (D.H. and C.E., personal observations), but none of our participants had experienced such exceptional practices, and this goes for all three groups.

## 5 Conclusion

The significance of this exploratory study lies in its minute description of the motor repertoire of infants with Down syndrome aged 3–5 months. During this time window, fidgety movements are the predominant spontaneous movement pattern and an excellent marker for the neurological outcome (Bosanquet et al., 2013, Einspieler et al., 2004, Einspieler et al., 2016). Infants with Down syndrome already show motor impairments at this early age, as evidenced by a significantly low MOS. Reassessing the same children as toddlers will show whether the high predictive power of fidgety movements found in infants with and without acquired brain injury (Bosanquet et al., 2013, Einspieler and Prechtl, 2005, Einspieler et al., 2004, Prechtl et al., 1997) also holds true for infants with a genetic disorder. A particularly important aspect will be whether the early spontaneous motor repertoire will also assist to predict the cognitive development of individuals with Down syndrome. A particularly important aspect that needs to be studied is the predictive power of the early spontaneous motor repertoire for the cognitive development of individuals with Down syndrome. But quite apart from this important aspect for clinicians and researchers, atypical postural features such as external rotation and abduction of the hips and/or internal rotation and pronation of the wrists call for earlier intervention.

## Appendix A

GenGM Study Group: Fernanda F. Genovesi (São Paulo), Renata Hydee Hasue (São Paulo), Magdalena Krieber (Graz), Zan Mustacchi (São Paulo), Anna Scheuchenegger (Graz), Xiu-shu Shen (Shanghai), Iris Tomantschger (Graz), Berndt Urlesberger (Graz), Bilge N. Yardımcı (Ankara), Dajie Zhang (Graz).
